# Evaluation of fecal *Lactobacillus* populations in dogs with idiopathic epilepsy: a pilot study

**DOI:** 10.1186/s42523-020-00036-6

**Published:** 2020-05-11

**Authors:** Karen R. Muñana, Megan E. Jacob, Benjamin J. Callahan

**Affiliations:** 1 Department of Clinical Sciences, College of Veterinary Medicine, North Carolina State University, Veterinary Health Complex Room 2569, 1052 William Moore Drive, Raleigh, NC 27607 USA; 2 Department of Population Health & Pathobiology, College of Veterinary Medicine, North Carolina State University, Raleigh, NC 27607 USA

**Keywords:** Seizures, Clinical study, Canine, Gut-brain-axis, Gut microbiota, Dysbiosis, Antiepileptic drugs

## Abstract

**Background:**

Idiopathic epilepsy is a common neurological disorder of dogs characterized by recurrent seizures for which no underlying basis is identified other than a presumed genetic predisposition. The pathogenesis of the disorder remains poorly understood, but environmental factors are presumed to influence the course of disease. Within the growing body of research into the microbiota-gut-brain axis, considerable attention has focused on the protective role of Lactobacilli in the development and progression of neurological disease. Investigations into the association between the gut microbiome and epilepsy are in their infancy, but some preliminary findings support a role for Lactobacilli in drug resistant epilepsy. To date, there are no published studies evaluating the gut microbiome in dogs with epilepsy. This pilot study was undertaken to evaluate fecal *Lactobacillus* populations in dogs with idiopathic epilepsy compared to healthy dogs.

**Results:**

Fecal samples were obtained from 13 pairs of dogs, consisting of a drug-naïve epileptic dog and a healthy dog from the same household and maintained on the same diet. Evaluation of large-scale microbial patterns based on 16S rRNA gene amplicon sequencing identified a household effect in the study population. Differential prevalence testing at the 16S rRNA gene sequence variant and genus levels did not identify any statistically significant differences between epileptic and control dogs. Quantitative PCR of *Lactobacillus* species isolated through culture revealed no statistically significant difference between the epileptic and control dogs (median concentration, 3.8 log_10_ CFU/g feces and 4.6 log_10_ CFU/g feces, respectively). *Lactobacillus* in culture was not killed by exposure to phenobarbital, potassium bromide, zonisamide, or levetiracetam.

**Conclusions:**

This pilot study did not identify any difference in large-scale microbial patterns or relative or absolute abundance of *Lactobacillus* species in drug-naïve epileptic dogs compared to healthy dogs. Further studies are warranted to evaluate the role of the gut microbiome in disease progression and treatment response in dogs with epilepsy. Lactobacilli in culture were not killed or inhibited from growing when exposed to phenobarbital, potassium bromide, zonisamide or levetiracetam, suggesting that antiepileptic drug administration is less likely to be a confounding factor in future studies evaluating the role of *Lactobacillus* in epilepsy.

## Background

Epilepsy is the most common chronic neurological disorder of dogs [1], with an estimated prevalence of 0.75% in the general population [2]. Approximately half of affected dogs are diagnosed with idiopathic epilepsy, a clinical syndrome characterized by recurrent seizures for which there is no underlying cause other than a presumed genetic predisposition [3–5]. Despite its significance to canine health, idiopathic epilepsy remains a clinical diagnosis of exclusion, underscoring the fact that its pathogenesis is poorly understood. Idiopathic epilepsy is presumed to be a complex, polygenic disorder in most affected dog breeds [6], that is likely influenced by environmental and developmental factors [7].

Gastrointestinal health figures prominently among the environmental factors that have been proposed to influence the course of epilepsy. An association between gastrointestinal disease and epilepsy is well established in humans. Recent meta-analyses have identified a bidirectional relationship between epilepsy and celiac disease [8, 9], with improved seizure control observed in affected patients following the implementation of a gluten free diet [10]. Furthermore, population-based studies have identified a greater risk of epilepsy in adults with inflammatory bowel disease as well as irritable bowel syndrome [11–13]. Although a similar association has yet to be demonstrated in dogs, it is a firmly held belief among caregivers of dogs with epilepsy that diet and gastrointestinal health can influence the course of disease. This was demonstrated in a recent web-based survey in which 68% of respondents reported changing their dogs’ diet after receiving the diagnosis of idiopathic epilepsy, and 20% reported administering probiotic or prebiotic products to their epileptic dog as an aid in the management of the disorder [14].

There is a growing body of research into the microbiota-gut-brain axis and its role in health and disease. Considerable attention has focused on the beneficial effects of *Lactobacillus* species, a presumed gastrointestinal symbiont constituting less than 1% of the total bacterial community within the fecal microbiota of both humans [15] and dogs [16]. Alterations in Lactobacilli populations in the gut have been linked to the development and progression of several neurological conditions, including anxiety/depression [17, 18], autism spectrum disorder [19], multiple sclerosis [20] and Alzheimer’s disease [21]. In addition to possessing anti-inflammatory properties [22], *Lactobacillus* species can produce the inhibitory neurotransmitter gamma-aminobutyric acid (GABA) [23], and increased GABA concentrations in the gastrointestinal tract has been shown to correlate with increased levels in the central nervous system [24]. The notion that gut *Lactobacillus* can influence brain function through the modulation of GABA was persuasively demonstrated in a study in which healthy mice were chronically administered oral *Lactobacillus*, resulting in increased expression of GABA receptors in the brain and an associated reduction in the level of anxiety and depression related behaviors. Furthermore, the effect was not observed in vagotomized mice, establishing the vagus nerve as a communication pathway between the gut and the brain [17].

Seizures arise from an imbalance in excitatory and inhibitory pathways in the brain that results in a hyperexcitable, hypersynchronous neuronal state. Consequently, it seems plausible that *Lactobacillus* could serve a protective role in the development and progression of epilepsy as a result of its ability to alter brain activity through an increase in GABA-mediated inhibitory neurotransmission. This hypothesis has been explored in a few recent studies involving rodent models of epilepsy [25] as well as naturally occurring disease in humans [26, 27], with promising results. Our pilot study was undertaken to evaluate the gut microbiota, with a focus on Lactobacilli, in dogs with idiopathic epilepsy. Our aims were to 1) determine differences in higher-order microbiota populations in the feces of drug naïve epileptic dogs when compared to controls; 2) identify and quantify the *Lactobacillus* species in the feces of drug naïve epileptic dogs when compared to control dogs; and 3) determine whether the growth of Lactobacilli is affected by antiepileptic medication.

## Results

### Animals

Twenty-six dogs, consisting of 13 epileptic dogs each paired to a healthy dog from the same household, were included in the study (Table 1). Fecal samples were collected from 28 dogs (14 pairs); however, 1 of the samples was of insufficient quantity for the study protocol, and that pair was excluded from any further analysis. Breeds represented included Siberian Husky, Belgian Tervuran, Australian Shepherd, Rough Coated Collie, Shetland Sheepdog, Havanese, Border Terrier, German Shorthaired Pointer, Golden Retriever, Welsh Springer Spaniel and mixed breed. Both dogs were of the same breed in 11 of 13 (85%) pairs, and 3 of these pairs were known to be related. Age of dogs ranged from 1 to 11 years, with a median of 6 years. The study population consisted of 11 females (8 spayed) and 15 males (12 neutered). There was no statistically significant difference in age or sex between the epileptic and control groups.

**Table 1:** Summary of demographic characteristics of paired epileptic and control dogs

Epileptic dog			Control dog		
Breed	Age (years)	Sex	Breed	Age (years)	Sex
Siberian Husky	3	MN	Siberian Husky	3	F
Siberian Husky	4	FS	Siberian Husky	8	FS
Australian Shepherd	6	MN	Australian Shepherd	10	MN
Rough Coated Collie	2	MN	Rough Coated Collie	6	MN
Mixed breed	6	FS	Mixed breed	6	MN
Belgian Tervuren	3	F	Belgian Tervuren	7	FS
Belgian Tervuren	8	M	Belgian Tervuren	9	F
Shetland Sheepdog	5	MN	Shetland Sheepdog	6	FS
Mixed breed	1	M	Mixed breed	3	MN
Havanese	5	FS	Havanese	4	MN
Border Terrier	11	MN	Border Terrier	10	MN
Golden Retriever	8	FS	Welsh Springer Spaniel	4	M
German Shorthaired Pointer	1	FS	German Shorthaired Pointer	8	MN

*F* Female intact, *FS* Female spayed, *M* Male intact, *MN* Male neutered

### 16S rRNA gene amplicon sequencing

**Fig. 1:**
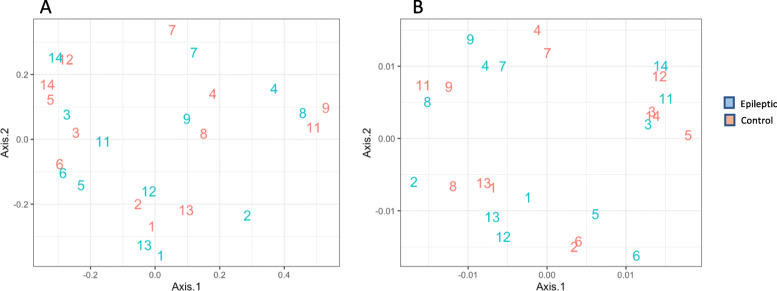
Depiction of the similarity between the large-scale microbial patterns in study dogs using the Bray-Curtis (**a**) and weighted unifrac (**b**) distance metrics. Each number represents a household. In these analyses, the more similar the community pattern, the closer in proximity the data points are located. The same numbers in red and blue are seen to be in relative close proximity to one another, confirming that there is a strong household effect (Permanova, *P* < 0.001)

Evaluation of large-scale microbial patterns in the population of dogs based on community wide distances (Fig. 1) demonstrated a clear household effect (Permanova, *P* < 0.001). Neither epileptic status, age, nor sex were significantly associated with community composition (Permanova, *P* > 0.05). Seven different 16S rRNA gene sequence variants belonging to the *Lactobacillus genus* were detected, each of which was assigned to an individual *Lactobacillus* species or a pair of *Lactobacillus* species when those two species were identical over the sequenced gene region. The prevalence of the different *Lactobacillus* sequence variants and their overall frequencies (relative abundances) across the entire study are summarized in Table 2. Differential prevalence testing of *Lactobacillus* at both the 16S gene sequence variant and genus levels did not identify any statistically significant differences between the epileptic and control groups (Fisher’s exact test, *P* > 0.05).

**Table 2:** The prevalence of the different Lactobacillus sequence variants and their overall frequencies (relative abundances) in the study population identified through 16S rRNA sequencing

Species	Prevalence	Frequency
*Lactobacillus murinus/animalis*	14	0.043%
*Lactobacillus johnsonii/gasseri*	9	0.061%
*Lactobacillus reuteri*	6	0.051%
*Lactobacillus amylovorus*	5	0.0009%
*Lactobacillus casei/paracasei*	4	0.0042%
*Lactobacillus sakei/curvatus*	4	0.0011%
*Lactobacillus aviarius*	2	0.0015%

### *Fecal culture for identification of* Lactobacillus *species*

Of the 26 dogs in the study, at least one presumptive *Lactobacillus* species isolate was obtained from 22 dogs (85%). The number of isolates recovered from each dog ranged from 1 to 10. Sixty-one *Lactobacillus* isolates were positively identified by Sanger sequencing of the 16S rRNA gene, including 24 in the epilepsy group and 37 in the control group. Nine species were recovered, including *L. casei*, *L. animalis, L. murinus, L. apodemi, L. fermentum, L. gasseri, L. parabrevis, L. reuteri,* and *L. plantarum*. The mean number of identified isolates per dog in the epilepsy group (1.85; SD 1.6) did not differ significantly from that of the control group (2.85; SD 3.0).

### *Quantitative PCR (qPCR) of* Lactobacillus *species*

**Fig. 2:**
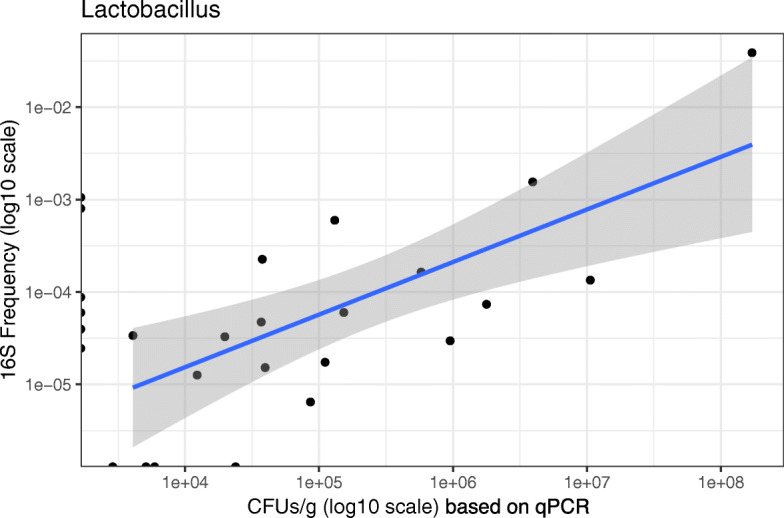
Comparison of the relative abundance of *Lactobacillus* from 16S rRNA gene sequencing to quantification based on culture and PCR, demonstrating reasonable agreement that is consistent with expectations given the differences between the methods (Student’s t-test *p* = 0.0013, R^2^ = 0.5334). The grey shaded area represents the 95% confidence interval around the linear regression line

Using qPCR of the *Lactobacillus* 16S rRNA gene, *Lactobacillus* species were quantified from the feces of 20 dogs. Six dogs had *Lactobacillus* concentrations below the level of detection (< 2.7 log_10_ colony forming units [CFU]/g feces) on qPCR; this included 4 dogs in the epileptic group and 2 dogs in the control group. For all dogs in which *Lactobacillus* was detected, the quantity ranged from 3.7–8.2 log_10_ CFU/g of feces, with a median of 4.8 log_10_ CFU/g of feces. For the statistical analyses, samples with *Lactobacillus* concentrations below the level of detection were assigned a value of 2.7 log_10_ CFU/g feces, the lowest level of detection for the assay. The median concentration of *Lactobacillus* in the epileptic group was 3.8 log_10_ CFU/g feces, while the median concentration in the control group was 4.6 log_10_ CFU/g feces. No statistically significant difference in *Lactobacillus* concentrations between the epileptic and control dog pairs was identified. Comparison of the frequency of *Lactobacillus* species identified through the 16S rRNA amplicon sequencing and culture techniques yielded reasonable agreement (Fig. 2).

### Evaluation of the effect of antiepileptic drugs on the growth of Lactobacilli

**Fig. 3:**
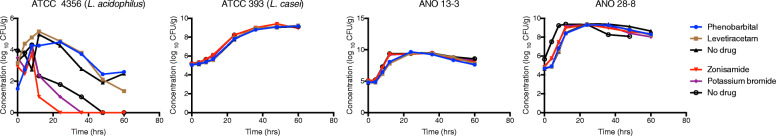
Concentration of *Lactobacillus* over time while incubated with therapeutic concentrations of phenobarbital (20 μg/mL), levetiracetam (20 μg/mL), zonisamide (20 μg/mL), and potassium bromide (2.0 mg/mL). Four strains of *Lactobacillus* were evaluated for each drug, two obtained from the ATCC (ATCC 4536, *L. acidophilus* and ATCC 393, *L. casei*) and two anonymized samples obtained from dogs in the study (ANO-13-3 and ANO 28–8). Incubation with no drug served as the positive control

*Lactobacillus* were not killed or inhibited from growing by therapeutic concentrations of any of the compounds. One *Lactobacillus* strain (ATCC 4356; *L. acidophilus*) was inhibited or killed during each assay (all antiepileptic compounds as well as the positive growth control) and should be considered an outlier based on growth behavior. All other strains proliferated when exposed to the various drugs, with no strain demonstrating a reduction in *Lactobacillus* concentration > 3 log_10_ CFU/mL between any two time points (Fig. 3).

## Discussion

In this pilot study, we evaluated the fecal microbiome in dogs with epilepsy utilizing 16S  rRNA high throughput gene sequencing and culture techniques, and did not identify any statistically significant difference in either large-scale microbial patterns or the relative or absolute abundance of *Lactobacillus* species compared to healthy dogs. However, an alternative explanation for these negative results is the low power due to the small size of this pilot study. We also determined that the growth of Lactobacilli in culture is not altered by phenobarbital, potassium bromide, zonisamide or levetiracetam, 4 antiepileptic drugs commonly used in veterinary practice.

The gut microbiome in dogs is known to be influenced by genetics [28] as well as environmental factors such as habitat [28, 29] and diet [30–34]. Our study was designed to compare pairs of dogs from the same household and fed the same diet, in order to minimize the effect of these confounding environmental factors. We did not attempt to control for genetics, but coincidentally 11 of the 13 pairs of dogs were of the same breed. Evaluation of large-scale microbial patterns in the population of dogs identified a strong household effect, thereby supporting the validity of the study design. Age and sex are additional factors that have been shown to be associated with variation of the microbiome in other mammalian species [35]. Results from a limited number of studies support an effect of age but not sex on the gut microbiome in dogs [28, 29]. There was no effect of either age or sex on large-scale microbial patterns in the population of dogs evaluated in this study. However, the dogs were within a fairly narrow age range, with > 70% being 4–8 years of age, and this could have contributed to the lack of an identifiable age effect. Previous studies that have identified age-related variations in the gut microbiome of dogs have either focused on juvenile dogs 7 weeks-18 months of age [28], or involved adult dogs that were more equally distributed over 1–12 years of age [29].

Drug-naïve epileptic dogs were recruited for this pilot study, in order to mitigate any effect of administered drugs and to explore whether disease status alone was associated with alterations in the gut microbiome. No statistically significant difference in large scale microbial patterns were identified between the epileptic and control dog populations, providing no evidence that gut dysbiosis is associated with the development of epilepsy. Idiopathic epilepsy is a heterogenous disease, with individual differences noted in disease severity and course of progression [3], and the inclusion of only drug-naïve dogs likely selected for a population with a relatively mild form of epilepsy. Only 2 of the epileptic dogs enrolled in the study were reported to have a history of cluster seizures. A longitudinal study of dogs with new onset seizures demonstrated that antiepileptic drug treatment is more likely to be initiated in dogs with a history of cluster seizures compared to dogs that experience isolated seizure episodes [3].

Over the last decade, there has been extensive research into the gut-brain-axis and the role of the gut microbiome in neurological disease. However, investigations into the association between the gut microbiome and epilepsy are in their infancy, with published reports only emerging over the last few years. A 2018 study in humans evaluated the fecal microbiome in epilepsy patients compared to healthy family members. Epileptic patients were grouped based on whether seizures were drug-sensitive or drug-resistant, according to the International League Against Epilepsy guidelines [36]. The study demonstrated that drug-sensitive epileptic patients had fecal microbial compositions that were similar to healthy controls. However, patients with drug-resistant seizures had significant alterations in the gut microbial populations, with increased abundance of bacteria that are typically considered rare [26]. In a separate study into the mechanism of action of the ketogenic diet as a treatment for refractory epilepsy, investigators identified significant alterations in the gut microbiome of rodents fed a ketogenic diet, and demonstrated that these changes were required for the diet’s anti-seizure effect [37]. Results of these studies suggest that the gut microbiome may play a role in drug resistance in epilepsy, and hence may be more likely to influence disease course and treatment response rather than disease development.

Furthermore, there are a few recent studies to support a potential beneficial effect of *Lactobacillus* in the management of drug resistant epilepsy. Using chemically kindled rats as a model of drug resistant epilepsy, investigators demonstrated that oral administration of a *Lactobacillus-*containing probiotic was associated with a significant decrease in epileptic activity. Rats that received the supplement had elevated concentrations of GABA in brain tissue compared to controls [25], supporting the premise that *Lactobacillus* might influence seizure control through alterations in inhibitory neurotransmission. A clinical study evaluating the fecal microbiome in 91 humans with epilepsy demonstrated increased abundance of *Bifidobacteria* and *Lactobacillus* in patients with 4 or fewer seizures a year compared to patients with more than 4 seizures a year, suggesting that these bacteria might play a protective role in epilepsy [26]. An open-label, pilot study involving 45 humans with drug resistant epilepsy reported that 29% of patients experienced a > 50% reduction in seizures after being administered a daily probiotic supplement containing *Lactobacillus* [27]. Additional research is needed to substantiate these findings and further evaluate the effect of *Lactobacillus* in the treatment of epilepsy.

Of the antiepileptic drugs utilized in veterinary practice, phenobarbital, potassium bromide, levetiracetam and zonisamide are used most commonly in dogs in the United States [38]. As part of this study, we explored the potential of these common antiepileptic drugs to influence the gut microbiota, and demonstrated no bactericidal effect on *Lactobacillus* in culture. Numerous classes of drugs, including non-antibiotics, can alter the composition of the gut microbiome, although no clear antimicrobial effect was demonstrated among 16 representative drugs of the class containing antiepileptics [39]. However, prenatal exposure to valproic acid has been shown to alter the gut microbiota in mice [40], and lamotrigine may inhibit microbial growth in culture by its effect on ribosomal biogenesis [41]. Moreover, gut microbes can alter the metabolism of antiepileptic drugs, as has been demonstrated for zonisamide [42] and clonazepam [43]. Potential interactions between the gut microbiome and antiepileptic drugs must be taken into consideration when exploring the role of the gut-brain-axis in the management of epilepsy.

The main limitation of this pilot study is the small sample size, that restricts the power of the study to detect potential differences between the groups. The use of dog pairs from the same household aimed to reduce confounding factors and improve the strength of the data. The study design was validated by the finding that household was the only factor determined to have a significant influence on large-scale microbial patterns, although genetics could explain some of this effect since most dog pairs in our study were also the same breed. Nonetheless, the study findings should be interpreted with the small sample size limitation in mind. In addition, our study hypothesis focused on fecal *Lactobacillus* populations, and therefore, detailed evaluation of other microbial populations was limited. Finally, the study enrolled only drug-naïve epileptic dogs, and as mentioned previously, these dogs were more likely to have a milder form of epilepsy. Consequently, the results cannot be generalized to all dogs with epilepsy.

## Conclusions

We determined that gut *Lactobacillus* populations in drug-naïve epileptic dogs are similar to healthy dogs from the same households. By evaluating drug-naïve dogs, this study focused on any potential influence of the gut microbiome on the development of epilepsy. Further studies are warranted to evaluate the role of the gut microbiome in the progression of epilepsy and treatment response in dogs. The finding that none of the antiepileptic drugs that were evaluated killed or inhibited the growth of *Lactobacillus* in culture suggests that antiepileptic drug administration is less likely to be a confounding factor in future studies evaluating the role of *Lactobacillus* in epilepsy.

## Methods

### Animals

Dogs were recruited in pairs, consisting of a dog with epilepsy and a healthy dog from the same household to serve as a control. To be included in the study, epileptic dogs were required to have a presumptive diagnosis of idiopathic epilepsy based on: 1) the presence of 2 or more unprovoked seizures occurring at least 24 h apart; 2) seizure onset between 6 months and 6 years of age; 3) normal interictal neurological exam; and 4) lack of laboratory abnormalities to suggest an underlying cause for the seizures. Epileptic dogs were not to have been administered any antiseizure drugs for at least 1 month prior to study participation. In addition, both dogs in a pair were required to be maintained in the same environment and be fed the same diet, not be administered any medications aside from parasite preventatives, and have normal appearing stool (as determined by the owner) for at least 2 weeks prior to enrollment and sample collection. Owners were required to provide informed consent prior to study participation. The study was approved by the North Carolina State University Institutional Animal Care and Use Committee.

### Fecal samples

Owners were asked to collect a fresh fecal sample free of ground contamination from each dog. Owners were sent collection and shipping materials, and were provided detailed, written instructions on sample collection, storage and shipment. Owners were instructed to refrigerate samples after collection, and ship the samples on ice packs to the investigator’s laboratory for next day delivery. In conjunction with sample submission, owners completed a brief online questionnaire on demographic information, diet, environment, and seizure history.

Upon receipt by the investigators, a routine fecal float screening for intestinal parasites was performed. Briefly, 2 g of feces was thoroughly mixed with 10 mL of FecaMed sodium nitrate solution (Vedco, Inc., St. Joseph, MO) using a wooden popsicle stick. The mixture was filtered through 1 layer of gauze and centrifuged for 5 min at 1300 rpm. After settling for 10 min, the top layer was removed, placed on a slide, coverslipped and evaluated under the microscope for the presence of parasite eggs. Any sample that tested positive for parasites was excluded from the study; owners were advised to have their dog treated for the parasitic infection and collect another fecal sample for inclusion in the study a minimum of 4 weeks later. Samples that were free of intestinal parasites were further aliquoted for the study; a portion of fresh feces was used for culture and qPCR, and the remainder of the sample stored at − 80 °C for further analysis.

### DNA isolation and 16S rRNA gene amplicon sequencing

Approximately 0.5 g of frozen fecal sample from each dog underwent DNA isolation and 16S rRNA gene amplicon sequencing through the Microbiome Core Facility at the University of North Carolina School of Medicine. To isolate DNA, samples were transferred to a 2 ml tube containing 200 mg of ≤106 μm glass beads (Sigma, St. Louis, MO) and 0.3 ml of Qiagen ATL buffer (Valencia, CA), supplemented with 20 mg/ml lysozyme (Thermo Fisher Scientific, Grand Island, NY). The suspension was incubated at 37 °C for 1 h with occasional agitation. The suspension was then supplemented with 600 IU of Qiagen proteinase K and incubated at 60 °C for 1 h. Finally, 0.3 ml of Qiagen AL buffer was added and a final incubation at 70 °C for 10 min was carried out. Bead beating was then employed for 3 min in a Qiagen TissueLyser II at 30 Hz. After a brief centrifugation, supernatants were aspirated and transferred to a new tube containing 0.3 ml of ethanol. DNA was purified using a standard on-column purification method with Qiagen buffers AW1 and AW2 as washing agents, and eluted in 10 mM Tris (pH 8.0). 12.5 ng of total DNA were amplified using universal primers targeting the V4 region of the bacterial 16S rRNA gene [44, 45]. Primer sequences contained overhang adapters appended to the 5′ end of each primer for compatibility with Illumina sequencing platform. The complete sequences of the primers were:

515F-5’TCGTCGGCAGCGTCAGATGTGTATAAGAGACAGGTGCCAGCMGCCGCGGTAA 3′.

806R-5’GTCTCGTGGGCTCGGAGATGTGTATAAGAGACAGGGACTACHVGGGTWTCTAAT 3′.

Master mixes contained 12.5 ng of total DNA, 0.2 μM of each primer and 2x KAPA HiFi HotStart ReadyMix (KAPA Biosystems, Wilmington, MA). The thermal profile for the amplification of each sample had an initial denaturing step at 95 °C for 3 min, followed by a cycling of denaturing of 95 °C for 30 s, annealing at 55 °C for 30 s and a 30 s extension at 72 °C (25 cycles), a 5 min extension at 72 °C and a final hold at 4 °C. Each 16S amplicon was purified using the AMPure XP reagent (Beckman Coulter, Indianapolis, IN). In the next step each sample was amplified using a limited cycle PCR program, adding Illumina sequencing adapters and dual-index barcodes (index 1(i7) and index 2(i5)) (Illumina, San Diego, CA) to the amplicon target. The thermal profile for the amplification of each sample had an initial denaturing step at 95 °C for 3 min, followed by a denaturing cycle of 95 °C for 30 s, annealing at 55 °C for 30 s and a 30 s extension at 72 °C (8 cycles), a 5 min extension at 72 °C and a final hold at 4 °C. The final libraries were again purified using the AMPure XP reagent (Beckman Coulter), quantified and normalized prior to pooling. The DNA library pool was then denatured with NaOH, diluted with hybridization buffer and heat denatured before loading on the MiSeq reagent cartridge (Illumina) and on the MiSeq instrument (Illumina). Automated cluster generation and paired–end sequencing with dual reads were performed according to the manufacturer’s instructions.

To analyze sequencing data, multiplexed paired-end fastq files were produced from the sequencing results of the Illumina MiSeq using the Illumina software configureBclToFastq. The DADA2 software was used to process the paired-end fastq files into a table of amplicon sequence variants (ASVs) observed in each sample [46]. Read pairs were truncated to forward and reverse lengths of 240 and 180, respectively. Read pairs were removed if the number of expected errors in the truncated forward or reverse read exceeded 2. ASVs were inferred using the DADA2 algorithm operating in pooled mode to increase sensitivity to rare variants. Chimeric sequences were identified and removed using the `removeBimeraDenovo` function. Taxonomy was assigned down to the genus level using the naïve Bayesian classifier method and version 128 of the Silva reference database. Species-level identification was made by exact matching against the Silva reference database using the `assignSpecies` function in the DADA2 R package. All exact species matches were reported for ASVs that exactly matched reference sequences from more than one species.

### *Fecal culture for identification of* Lactobacillus *species*

1 g of fresh feces was diluted into 9 mL of phosphate buffered saline (PBS) and homogenized. One hundred microliters of the sample was plated and streaked for isolation onto two Lactobacilli MRS agar plates (Difco, BD; Sparks, MD). One plate was incubated anaerobically for 72 h at 37 °C, and the other plate was incubated under 5% CO_2_ for 72 h at 37 °C. Five colonies per plate (10 per fecal sample) were selected and re-isolated for purification. After incubation, colonies were confirmed biochemically to be of the *Lactobacillus* genus (Vitek2 Compact; Biomeriux, Marcy-I’Etoile, France). Isolates were frozen in glycerol at − 80 °C and later sent to the North Carolina State University Genomic Science Laboratory for 16S rRNA sequencing to determine species.

### *Quantitative PCR (qPCR) of* Lactobacillus *species*

To more specifically quantify the *Lactobacillus* component of the fecal microbiota, genomic DNA extraction was performed on 250 mg of each fresh fecal sample using a standard kit (PowerFecal DNA Isolation Kit; MoBio Laboratories, Inc.; Carlsbad, CA). Extracted DNA was quantified using a spectrophotometer. A previously described qPCR with genus-specific primers was used to determine the concentration of *Lactobacillus* from each canine fecal sample [47]. A standard curve was generated using ten-fold dilutions of a cocktail of three previously characterized *Lactobacillus* strains (*L. rhamnosus* ATCC 7469; *L. acidophilus* ATCC 4356; *L. delbrueckii* ATCC 11842) for comparison. Each sample was evaluated in triplicate.

### Evaluation of the effect of antiepileptic drugs on the growth of Lactobacilli

Four different *Lactobacillus* strains (two ATCC-type strains, and two strains randomly selected from epileptic study dogs) were utilized. Isolates were grown overnight on blood agar plates (Remel, Lenexa, KS). Colonies (approximately 5) were selected and inoculated into 5 mL of cation-adjusted Mueller Hinton Broth (MHB) and incubated at 37 °C under 5% CO_2_ to a 0.5 McFarland concentration. One milliliter of sample was diluted ten-fold in PBS, and 100 μL of the diluted sample was inoculated into 5 ml of MHB containing the desired concentration of antiepileptic drug. Four different antiepileptic drugs, obtained from the U.S. Pharmacopeial Convention, were evaluated at concentrations considered to be therapeutic (phenobarbital, 20 μg/mL; bromide, 2.0 mg/mL; levetiracetam, 20 μg/mL; zonisamide 20 μg/mL). A sample containing no drug was used as a positive growth control for all isolates. The tubes were incubated at 37 °C under 5% CO_2_ and at 0 h, 4 h, 8 h, 12 h, 24 h, 36 h, 48 h and 60 h post enrichment, 150 μl was removed, serially diluted ten-fold in PBS and plated in triplicate onto blood agar plates. Plates were incubated overnight at 37 °C under 5% CO_2_. Dilutions with between 10 and 100 colonies were counted to determine the concentration of *Lactobacillus* species at each time interval. All isolates for each treatment combination were run in duplicate. A negative control with no bacteria was used to confirm the assay remained free of contamination during the 60-h experiment. Differences in the growth of *Lactobacillus* in the presence of antiepileptic drugs were determined by comparing the mean colony count for each isolate replicate within a treatment group at all time points. Colony counts were transformed to log_10_ CFU/mL, and the average of each duplicate within each treatment calculated. Bactericidal activity was considered a reduction in *Lactobacillus* concentration of equal to or greater than 3 log_10_ CFU/mL (99.9% reduction) between two time points.

### Statistical analysis

Differences in sex and age between the epileptic and control group were evaluated using the Person’s chi-squared and Mann-Whitney test, respectively. Statistical analysis of fecal community composition was performed in the R statistical software environment (R) using the phyloseq R package [48]. Large scale microbial patterns were evaluated based on community wide distances using the Bray-Curtis and weighted Unifrac community dissimilarity measures, and visualized using multi-dimensional scaling. Statistical testing of the influence of sample covariates on community composition was performed using the PERMANOVA method as implemented in the VEGAN R package [49, 50], with permutations restricted to exchanging labels on dogs within the same household to control for the strong household effect. The association between the logarithm of *Lactobacillus* CFU abundances and the logarithm of *Lactobacillus* 16S rRNA gene frequencies was assessed using the lm function in R, excluding samples in which *Lactobacillus* abundance or frequency was measured to be zero. Differential prevalence testing was performed to evaluate for differences in epileptic and control dog populations using Fisher’s exact method. Differences in the concentration of *Lactobacillus-*specific 16S rRNA genes as determined by qPCR, and the proportion of different *Lactobacillus* species as determined by culture-based methods were compared between epileptic and control dogs using a paired t-test. A significance level of *p* < 0.05 was established for all analyses.

## Data Availability

The raw 16S rRNA gene sequencing data has been deposited in the SRA under the deposition PRJNA612483. The computational analyses that were performed are available in Rmarkdown format at the Github repository https://github.com/benjjneb/CanineEpilepsyManuscript

## Abbreviations

ASV: Amplicon sequence variants
CFU: Colony forming units
GABA: Gamma-amino butyric acid
MHB: Mueller Hinton broth
qPCR: Quantitative polymerase chain reaction
